# Perceptions of Kenyan adults on access to medicines for non-communicable diseases: A qualitative study

**DOI:** 10.1371/journal.pone.0201917

**Published:** 2018-08-24

**Authors:** Monica Adhiambo Onyango, Taryn Vian, Isabel Hirsch, Devashri D. Salvi, Richard Laing, Peter C. Rockers, Paul G. Ashigbie, Veronika J. Wirtz

**Affiliations:** 1 Department of Global Health, Boston University School of Public Health, Boston, Massachusetts, United States of America; 2 Faculty of Community Health Sciences, School of Public Health, University of the Western Cape, Cape Town, South Africa; University of Groningen, NETHERLANDS

## Abstract

In Kenya, noncommunicable diseases (NCDs) account for 27% of all deaths. Adult Kenyans have an 18% chance of dying prematurely from cancers, diabetes, cardiovascular diseases or chronic respiratory diseases. A Novartis Access Initiative is making medicines available to treat cardiovascular diseases, diabetes, chronic respiratory diseases, and breast cancer in 30 countries, including Kenya. Little is known about patients’ perceptions of access to medicines for NCDs in Kenya. The study objective was to understand patients’ perceptions of access to medicines; as well as barriers and facilitators at the household, community, and healthcare system level. A baseline qualitative study was conducted in eight of 47 counties as part of an evaluation of the Novartis Access Initiative in Kenya. The 84 patients interviewed through a household survey had been diagnosed and treated for an NCD. Although medicines at government facilities were free or cheaper than those sold in private pharmacies, the availability of medicines presented a constant challenge. Patients often resorted to private pharmacies, where NCD medicines cost more than at public facilities. Participants with an NCD took their health seriously and strove to get the medicines, even under difficult circumstances. Buying NCD medicines put a strain on the household budget, especially for the lower-income participants. Some actions to overcome affordability barriers included: borrowing money, selling assets, seeking help from relatives, taking on extra work, buying partial dosages, leaving without the medicines, or resorting to non-medical alternatives. In conclusion, access to NCD medicines is a major challenge for most adults in Kenya. As a result, they engage in complex interactions between public, private facilities and pharmacies to overcome the barriers. The government should ensure well-stocked public sector pharmacies and subsidize prices of medicines for lower-income patients. Integration of industry-led access to medicine programs may help governments to obtain low cost supplies.

## Introduction

While global life expectancy from birth increased from 61.7 years in 1980 to 71.8 years in 2015, deaths from NCDs such as cardiovascular disease, cancers, chronic respiratory diseases, and diabetes have steadily increased [1]. Over 86% of premature deaths from NCDs occur in low- and middle-income countries [2]. NCDs were the leading cause of life lived with disability in 94 of 138 countries [3]. Interventions for the prevention and control of NCDs are influenced by many factors, including the availability of affordable technologies and essential medicines for treatment in the public and private sector. The international community has urged pharmaceutical companies to promote access to essential medicines, and they have responded with many new initiatives [4, 5, 6].

In Kenya, NCDs account for 27% of all deaths, and people have an 18% chance of dying prematurely from cancers, diabetes, cardiovascular diseases or chronic respiratory diseases [7]. Kenya faces challenges in NCD control and treatment programs due to workforce shortages, frequent stock-outs of medicines, and problems guaranteeing the quality of medicines [8, 9]. Patients often have to pay for medicines, and out-of-pocket payments can be high [10].

Mwai and Murithi [11] in their study found that compared to a household affected by communicable disease, NCDs are associated with a 23.17% reduction in household income. A recent study also reported that annual hypertension medication cost in Kenya for a patient may range from $26 to $230 and $418 to $987 in public and private health facilities respectively [12]. Health insurance in Kenya is offered by private companies, community based and microfinance insurance schemes and the national hospital insurance fund (NHIF). The NHIF is a state corporation and the largest national insurer in Kenya [13]. Although the NHIF is open to any Kenyan over 18 years of age, it is compulsory for those employed by the government and voluntary for the private and informal sectors [13]. Part of the benefits of NHIF include coverage for diagnosis, screening and treatment of most NCDs including asthma, diabetes, hypertension, respiratory diseases and Cancer. Although there are ongoing efforts by the government to increase its coverage, only about 20% of Kenyans had NHIF insurance coverage in 2014 [12]. Poor households and those living in rural areas are most likely not to have insurance, and this affects their access to NCD medicines. Little is known about how Kenyan patients confront and cope with problems concerning access and affordability of NCD medicines, or their perceptions about quality of medicines.

Novartis Access is a social business initiative designed to make available a portfolio of 15 medicines for cardiovascular diseases, diabetes, chronic respiratory diseases, and breast cancer. The program is being implemented in low- and lower-middle income countries, including Kenya. Governments and non-governmental organizations can purchase a basket of medicines at a price of $1 per treatment per month. As of December 2016, the company had made 124,433 monthly treatments available to 11,867 patients, with products available in Kenya, Lebanon and Ethiopia [14]. In Kenya, these medicines are provided through the Mission for Essential Drugs and Supplies (MEDS), a major pharmaceutical wholesaler in the country. The medicines are supplied to public and private health facilities.

This qualitative study was part of a larger mixed methods study conducted in Kenya to evaluate the impact of Novartis Access on the availability and price of NCD medicines included in the program portfolio and their therapeutic equivalents. The study was conducted at health facilities and households in counties targeted by the program in Kenya, using a cluster-randomized trial design, described elsewhere [15]. The primary aims of the study at the household level were to determine the impact of Novartis Access on availability of NCD medicines, prices paid for medicines, and household expenditures. The main purpose of this qualitative analysis was to understand patient’s perceptions of access to medicines; as well as barriers and facilitators at the household, community, and health care system level.

## Methods

### Sampling and selection of study participants

The study was conducted in eight of the 47 counties in Kenya. The study counties were selected based on their security, safety and consistency in purchasing medicines from MEDS. Through a two-stage sampling procedure, 794 households were randomly selected from the eight study counties. Each county consists of an enumeration area which is the smallest administrative unit, equivalent to a village. Sampling consisted of establishing ten enumeration areas (EAs) in each county. At each EA, ten eligible households were identified for the larger study. A household was deemed eligible if at least one member 18 years or older had previously been diagnosed and prescribed medicine for any NCD addressed by Novartis Access (e.g. diabetes, asthma, hypertension and breast cancer). Any other adult members in the household that met this criterion were also enrolled in the study. For this qualitative analysis, every fifth house in an EA was eligible for an interview after completion of the quantitative surveys. A total of 84 qualitative interviews were conducted.

Prior to data collection, a three-day training was conducted for all enumerators involved in data collection and management. Training was provided on human subject’s protection, research ethics, and qualitative methods. Semi-structured study guides were pre-tested by the study team and any necessary changes to the study guide were made and resubmitted to relevant ethical review boards for approval. All study guides were developed in English, translated in Kiswahili and then back-translated to English to ensure accuracy (S1 File & S2 File). The baseline qualitative study was the first of a series to be conducted in the evaluation, including at project midline and endline. The baseline study questions explored participants’ perceptions about NCD medicines availability and stock outs, affordability, perceived quality of medicines and barriers, and coping strategies. Interviews were tape recorded. The duration of the interviews ranged from 15 to 46 minutes.

### Data managements and analysis

Interviews were transcribed and, when necessary, translated from Kiswahili and other Kenyan languages to English. Two researchers read the typed interview transcripts separately for accuracy and completeness. The two researchers subsequently developed the initial codes individually and then compared the codes to reach a consensus. Coding was done continuously by two additional researchers throughout the analysis; they created new codes and dropped or combined codes as appropriate. The typed transcripts were imported into NVivo 11 qualitative data analysis software for coding and analysis. During the coding process, data was continuously reviewed, emerging patterns noted, and relationships between constructs and themes identified.

### Ethical considerations

The protocol was approved by the Institutional Review Boards at Strathmore University in Kenya and at Boston University Medical Center, USA. Additional written permissions for all foreign researchers were obtained from the Kenyan National Council for Science and Technology. Written informed consent was obtained for all participants at the start of the study. All participants in the household surveys were compensated for their time with 30 minutes of mobile phone airtime, valued at US$0.53.

## Results

### Theme 1: Availability

#### General perceptions

Most participants stated that medicines were not available at the government facilities, which forced them to buy the medicines at private facilities and pharmacies. Medicines such as analgesics and anti-malarial medicines were more available than NCD medicines.

The drugs that we can’t get from our hospital … are [those for] asthma, high blood pressure, diabetes… And maybe the other ones for these “big” diseases. Like cancer. You can’t get [those medicines]. Maybe you’ll find pain killers. (HH Makueni 3)

Most respondents could name the available non-NCD medicines more frequently or easily than NCD medicines. The most commonly mentioned non-NCD medicines were analgesics, antimalarial medicines and some antibiotics [e.g. paracetamol, ibuprofen, chloroquine and co-trimoxazole]. Although participants were able to identify the analgesics by name, they often identified the NCD medicines based on shape, size, color or packaging. Very few knew the names of the NCD medicines.

The tablets normally available are the ones called Brufen [ibuprofen] or Panadol [paracetamol]. Sometimes one is lucky to be given drugs… But Panadol are never out of stock. (HH Nyeri 13)

Diagnoses and practices varied by county and healthcare facility. Most study participants received a diagnosis and a paper prescription at a government health facility, then went to a private pharmacy to fill the prescription, or filled the prescription partly at the hospital pharmacy before completing it at a private pharmacy. Reasons stated for purchasing medications at a private pharmacy rather than a government hospital or dispensary were primarily related to lack of availability.

Sometimes you get sick and you go to (the public hospital), and you are told there are no drugs. Now that forces you to depend on your pocket. You go up to town, so that at least you get that drug. (HH West Pokot 2)

Following an NCD diagnosis, if the prescribed medicines are not available at the government health facility, providers might refer patients to buy their medicines from a specific private pharmacy or tell them to buy medicines from any pharmacy. Few participants however, described being directly referred by a provider to a specific private pharmacy. When it occurred, participants expressed suspicion about the provider receiving some benefits from the referral.

[Is there a specific pharmacy they tell you to go and buy from?] No, they (doctors) just tell you to go and buy this drug in the private pharmacy. They don’t tell you from which pharmacy. (HH Embu 10)

Those doctors own most of the private pharmacies. And as they send you, they know those drugs are where they’re sending you. And if [the medicines are] not there it’s you who will have problems. If you go somewhere else, you can’t argue with them. Because they were sending you to buy these drugs at a place they own. (HH Makueni 8)

In situations where providers do not recommend a specific pharmacy, the burden falls on the patient to find the private pharmacy and buy the medicines. Depending on how much money they have, they might be able to obtain only a partial dose. If they lacked the money or motivation to move around in search of medicines, they went home without the medicine. There were a few incidents in which participants did not find medicines at a local private pharmacy and had to go to a different town to buy medicines. Consequently, transport costs, and lack of time off from work were major factors that discouraged them from obtaining medicines. A few participants resorted to herbal medicine whenever they were not able to access their prescribed medicines.

We are helpless. At that moment we are really helpless because we have to have Ksh200 ($2 USD) to send someone to Nairobi. (HH Makueni 7)

Herbal medicine… Maybe use it as a supplement. Let us say I use cinnamon. The powder. It is used by all whether sick or healthy… At least I feel comfortable with that one. (HH Nyeri 12)

#### I simply want health

Once participants understood that they had an NCD, they described taking their health seriously and viewed medicines as a priority. For example, when asked about their perceptions of corrupt practices, such as the possibility that health providers may be selling medicine directly to patients without going through an established pharmacy, participants said that they were not concerned about whether it was right or wrong, as long as it gave them access to medicines.

My opinion (about medical practitioners selling medicines directly to patients) is that I simply want health. Even if you go buy drugs from such outlets, all I desire is good health for me (HH Kwale 7)

### Theme 2: Affordability

The majority of participants stated that medicines are not affordable for the average person, either as a result of high prices, low income, or a combination of the two. In addition, most perceive that medicine prices have increased, and that medicines are more expensive today than in the past.

I am a subsistence farmer and my spending is on the higher side considering my income, which is little. (HH Kakamega 3)

I have been trying to keep money for treatment because [with] my sickness I realized I will live

with my disease for the rest of my life. (HH Makueni 4)

Most participants acknowledged that living with an NCD places an extra financial burden on their household. They explained that the financial burden of budgeting and purchasing medicines that are taken every day is high. A participant explained below:

The problem I have as a patient, the big challenge in paying for everything, it’s not that I pay today, and not tomorrow, I pay daily. I’ll be sick today and tomorrow. And there is no money. That’s where my problem is. (HH Makueni 3)

#### Prices of medicines vary

The majority of participants stated that prices vary for medications between facilities and private pharmacies. Some participants expected to receive medicines at no cost from government facilities, but most expected to pay for all or part of their medicines obtained from a government or private facility. Most participants mentioned that medicines at government facilities were free or cheaper than at private facilities. Some participants expect to pay some amount of money at the government facilities and buy the rest of medicines at a private pharmacy due to low availability at government facilities.

Yes, I was given medicines in the hospital (for free). But not many. I was told it was because the medicine has not arrived. When they are finished, I buy others. (HH Nyeri 2).

The high price of medicine is a major challenge echoed by most study participants. Participants had different ideas about why medicines are sold at different prices in different facilities. Many acknowledged that private pharmacies have more control over their prices because they are owned by individuals rather than the government.

Because, you know, the private pharmacy is owned by an individual. He sells drugs at his own pleasure. But the public one, owned by the government, they at least have mercy on us. (HH Samburu 2)

Given that prices are considerably different from facility to facility, some participants make a concerted effort to find the lowest priced medications. The participants lamented that it was difficult to establish where to get low-cost medicines. Participants therefore visit more than one pharmacy in order to find the least expensive medication.

Sometimes you are sent to a place where you can’t afford to buy the drugs. So one has to move from place to place and find the cheapest place to buy from. (HH Nyeri 13)

#### Paying for medicines

To pay for their medicines, participants borrow or accept money from their relatives, adult children or neighbors; arrange with pharmacy staff to buy their medications through a credit system; take odd jobs to increase their income when they need to buy medicine; use their salaries, health insurance or retirement funds; or sell household items such as crops or farm animals (e.g. cattle and chicken).

When I have no income at all, I can sell a chicken or sell a goat… So that I can meet the required money… Because I have to take care of the sickness… I don’t wait… I should not compare that chicken or goat with sickness. (HH Makueni 5)

I mostly (depend) on my children in Nairobi. If they back out [stop buying medicines for me], then it is the end of me. (HH Makueni 1)

Where I go to pick up these drugs, I get them even if I don’t have the cash. [Later,] when I get the money, I pay for them. (HH Samburu 7)

I pay using my National Health Insurance Fund (NHIF) card. They deduct a lot of money for NHIF. (HH Samburu 1)

#### Barriers to NCD medication access

While participants mentioned the availability of medicines as a factor, money is the primary barrier to medicine access. Participants mentioned the need to pay the fare for transportation to a pharmacy as well as the high prices of the medicines itself.

It is only money that will stop me from buying the drugs, if I have the money I will buy. (HH Kakamega 10)

At times I find that I don’t have money for transport- for going and for coming back… I lack transport. Lack of money is my biggest reason (for not acquiring medicines). (HH Nyeri 9)

As mentioned earlier, when participants are unable to buy their medications, they will often only fill part of their prescription until they can find the money for the full prescription. Otherwise, they will return home and wait until they can find the money, or resort to non-medical and herbal alternative treatments. Some of the actions taken when they are not able to afford the full prescription include buying partial dosage according to available finances, leaving without medicines, trusting in divine grace, and resorting to non-medical alternatives.

It’s a challenge but what can I do? I buy according to what I can afford. Maybe I can buy for one or two days, but when I travel to Nairobi I buy for the whole one or two months since medicines are cheaper there… [When I am here at home] I just buy for a week. (HH Narok 4)

I just drank hot water since I had no money to buy drugs. I went to the government hospital which is free but they did not give me even drugs. They never gave me drugs that could help me. (HH Kwale 8)

Effect on Household Expenditures: Participants reported using a large portion of their income to purchase medicines, and often stated that they have had to forgo buying necessary items in order to be able to buy medications. Participants mentioned having neglected to buy sugar, salt, flour, vegetables, pay school fees, or purchase animal feed in order to have enough money to buy medications.

Maybe the only 100 Ksh ($1 USD) I had in my pocket was supposed to buy flour… now I will be forced to buy drugs… because if I buy flour and then eat… then maybe at night my blood pressure increases… now you see my people will be troubled… So even my cow can sleep hungry or stay without anything… (I would buy medications over feeding) a chicken, assuming I had one. (HH Nyeri 10)

It’s better that someone sleeps without food, rather than miss drugs. (HH Samburu 6).

Effects on Health: Many of the tactics that participants reported that they use to be able to purchase medicines, have a detrimental effect on their health. Specifically, participants mentioned that taking odd jobs when one is feeling sick, and forgoing needed food purchases can negatively affect their health. In addition, participants commonly mentioned partial dosing or missed doses of medicines as adversely affecting their health.

Many people are sick at home and they almost succumb to death since they can’t afford the drugs. One is told to buy drugs, and since he/she doesn’t have money, they don’t take drugs. (HH Nyeri 13)

### Theme 3: Perceptions of quality

#### Attributes of good and bad medicines

The main attribute that most participants used to gauge the quality of a given medicine was whether or not it worked for them. The most common perception was that if it makes you feel better, the drug is of a good quality. If there was no improvement in their condition, the medicine was thought to be of poor quality.

You take the drug and you feel your body is fine. … that is, you get to know that this medicine is of good quality. (HH Kakamega 10)

There are some drugs you can take and your condition remains the same. Those drugs don’t help you. (HH Kwale 1)

When asked whether quality varied among different brands of medicines, most people said that they could not tell two brands apart. A participant from Nyeri mentioned, *“I have never been able to differentiate (between) the companies (HH Nyeri 12)*.*”* When participants were asked if the origin or location of where the medicine was manufactured had an impact on quality, most people did not know where their medicines were manufactured, but a few thought that imported medicines were of better quality.

Many of these participants thought that generic medicines are not as effective as those marketed by the innovator company. Due to this perception, some participants reported increasing their own dose without asking for advice from their doctor.

They are good. But these generic ones (are bad)… that's why I've said at times I take 3, or I double the dose. So it depends. Like me, I have had this disease so long that I know if I take generic, unless I double the dose, I will not feel better. (HH Kwale 2)

Most participants viewed doctors as the experts because of their knowledge of medicines. Questioning the doctor regarding the prescription or the quality of a medicine was not the norm.

They (doctors) are the people who know because they have learned (about the quality of medicines). They know this one is of good quality and this one is of poor quality. They have experience. (HH Nyeri 4)

You know, we do not ask such questions (about the quality of medicines) … You just go, state your problem and get an injection. (HH West Pokot 1)

## Discussion

### Availability

Our findings show that among the participants interviewed, most were diagnosed with an NCD at a government health facility, but went on to purchase their medicines elsewhere. Most government facilities did not always have the NCD medicines available, and the majority of participants bought their medicines at private pharmacies. There were instances in which the patients had to travel to more than one private pharmacy to find an affordable price for their NCD medicine. Some of the respondents ask relatives living in Nairobi or other towns/cities to purchase and send medicines to the patient.

Most concerning is that when medicines are unavailable, patients sometimes go without taking them for days or take partial dosages. This can have negative implications for their health in terms of disease progression, increased morbidity and mortality, and costs [16]. Unavailability of NCD medicines disproportionately impacts poorer patients because most often they cannot afford to travel in search of cheaper medicines due to the high cost of transportation, for example. Poorer patients are also more likely to be unable to send relatives to purchase medicine in other towns/cities. As a result, they have to purchase the medicines at the closest private pharmacy, which commonly is the most expensive option because it may be the only pharmacy in town and with the specific type of NCD medicine needed.

Our findings support research that documents the lack of availability of NCD medicines in Low and Middle Income Countries (LMIC) [17]. Most participants in our study also stated that medicines for infections or pain treatment were readily available compared to NCD medicines, specifically analgesics and anti-malarial medicines. These findings confirm previous research that has shown below required level availability of essential NCD medicines in government facilities compared to medicines used for acute conditions in developing countries [10, 17, 18]

Notably, most of the respondents in our study could not identify the NCD medicine that they were taking by name. They differentiated their medicines by shape, size, color or packaging. In contrast, participants knew the names of medicines used for other conditions such as infections and treatment of pain. These responses may have been obtained because the question simply asked patients to name medicines that are available at health facilities, and did not specifically ask for the names of NCD medicines. Even so, these responses corroborate participants’ perceptions that NCD medicines are commonly unavailable while medicines used for acute or symptomatic conditions such as analgesics are usually in stock at the government health facilities.

### Affordability

Patients explained that medicines were often cheaper and sometimes given out at no cost at the government health facilities. Because the government facilities do not always have the medicines available and patients resorted to private pharmacies, the majority of patients lamented that medicines were very expensive. As a result, buying NCD medicines put a strain on the household budget, especially for the lower-income participants. This pressure was confirmed by the actions the participants took to ensure that they could buy medicines: borrowing money, selling assets, seeking help from relatives, and taking on extra work.

Because NCDs are chronic, patients have to take medicine regularly. However, the challenges that patients encountered with medicine access created durations of time in which the poorer participants could not afford to purchase the medicines at all, and had no other options available to them. These findings are in line with a recent study in Kenya showing that the diagnosis and treatment costs of NCDs both in public and private sectors represent a substantial economic burden to patients [12], and that NCDs pose significant economic losses and poverty on Kenyan households [11]. Earlier studies also found that in most LMICs, the highest component of household health-related expenditure is on medicines, which leads to impoverishment because of high spending on essential NCD medicines [19]. Lack of health insurance often makes NCD medicines inaccessible and unaffordable to many populations in LMICs, where the prevalence of NCDs is increasing rapidly [10, 20].

Challenges confronting access to medicines in low and middle-income countries include weaknesses in health systems, fragmented and weak medicine supply and distribution systems, and financing the demand for chronic NCD medicines [19]. Lower-income patients bear the burden of NCDs and have to pay out of pocket for medicines. To make things worse, as confirmed by the participants, prices of medicines are not well regulated in Kenya. Private pharmacies therefore are free to charge any price, which is often marked-up. Because of this lack of regulation, ensuring the availability of generic NCD medicines in the public sector is likely to be the most cost-effective option to improve access to medicines [21]. This aligns with the goals of the Novartis Access Program, which aims to make available a package of NCD medicines in 30 LMICs countries, including Kenya.

Despite the struggle with the difficulty of balancing high prices and availability of NCD medicines, the respondents took their NCD condition very seriously once they were diagnosed. They made it a priority and a commitment to obtain the medicines, even under difficult circumstances. The patients demonstrated an interest in taking care of themselves, doing “whatever it takes” to get the NCD medicines. Most participants acknowledged that taking partial dosages and alternative remedies are not optimal choices, and viewed them generally as a last resort. These findings are similar to findings in studies of HIV adherence, which have shown that structural, rather than motivational factors are more important in predicting adherence [22].

### Quality

Respondents’ perceptions of quality were connected to whether the medicine made them feel better. They also relied on their doctor to choose the medicines for them. A few participants viewed imported medicines more favorably compared to medicines produced in Kenya. A majority of the participants preferred the type of medicines that they routinely use. Some even mentioned that a change of medicines made them feel sick and therefore it was rare for their medicines to be changed.

## Conclusion

The common barriers to NCD medicine access for most adults in Kenya include lack of medicines at government health facilities, money for medicines, and lack of money for transportation to a private pharmacy or facility to purchase medicines. To cope with lack of availability and affordability of medicines, most patients resort to buying partial doses of medicines, leave without medicines or resort to non-medical alternatives. These options can have detrimental outcomes on patients’ health. Government should ensure that government sector pharmacies are well-stocked, and should subsidize prices of medicines for lower-income patients. Integration of industry-led access to medicines programs may help governments obtain low-cost supplies. Patients should be educated to understand more clearly the names and types of NCD medicines that they are taking to overcome access barriers and increase adherence.

## Supporting information

S1 File (DOCX)

S2 File (DOCX)
