# Supplementary material for: Perceptions of Kenyan adults on access to medicines for non-communicable diseases: A qualitative study
Source: PLoS One. 2018 Aug 24;13(8):e0201917. doi: 10.1371/journal.pone.0201917 (PMC6108464; doi:10.1371/journal.pone.0201917)
Supplement: S1 File — (DOCX) [file pone.0201917.s001.docx]

**Instrument ID: H-KI**

Novartis Access Initiative

Household Key Informant Instrument

Target Audience:

1. Household adult with chronic illness

**Instructions for the Interviewer**

**Step 1:** **Informed Consent:** *Ask the participant for a few minutes of their time. Introduce yourself and the study. Begin the informed consent as per the training. If consent is granted, leave the informed consent sheet with the participant.*

**Was informed consent obtained?**

**YES ________ (proceed with interview)**

**NO** ________ (**STOP! Thank the participant for their time but do not proceed with the interview)**

***Interviewer****: Read the following statement. Please repeat the statement translated into the local language based on primary languages.*

“Thank you for agreeing to participate in this interview. My name is ______. I will be asking you the questions. My partner ______ will be taking notes on the things you have to say.

We are collecting baseline data on access to medicines in anticipation of a new initiative starting soon to improve access. We want to understand your views on medicines availability and what happens when medicines are not available. We would also like to ask your perceptions about the affordability and quality of medicines, and how you learn about and share information about medicines. Please feel free to tell us whatever you are comfortable sharing. You should also remember that you do not have to share anything that you are not comfortable sharing. There are no right or wrong answers, so please be honest and help us to understand what is true for you and your community. Are you ready to begin?”

**Step 2***: Please begin the interview with the demographic questions*.

**Step 3:** *Proceed to the Semi-structured interview guide. Please probe to obtain as in-depth and specific information you can. NOTE: If there is a spontaneously reported adverse event, please report it to the Principal Investigator [fill in appropriate contact information for PI or designate here]*

**Interviewer ID** _________ **Note-taker ID:________________**

1. **Interview Date (DD/MM/YYYY)** __________________
2. **Time Start** _______
3. **Supervisor initials __________**

**Table 1: Respondent Demographics**

***Interviewer:*** *“I’m going to start by asking you questions about yourself.”*

| Q# | QUESTION | CODE | | Response | SKIP |
| --- | --- | --- | --- | --- | --- |
| 000. | Respondent/Instrument type | H-KI: **(1)** | | **1**  _____________ |  |
|  | Respondent gender | Male  **(1)**  Female (**2)** | | _____________ |  |
|  | Age |  | | _____________ |  |
| 102. | Phone number |  | | ____________ | |
| 103. | Name of closest health facility |  | | ____________ | |
|  | Novartis Access Initiative is a program designed to increase availability and affordability of high quality medicines for several chronic diseases.  Had you ever heard of Novartis Access before now? | | No (**0)**  Yes (**1)** | __________ |  |

**Semi Structured Interview Guide: *Interviewer:*** *“Now I’m going to ask you questions about your ideas and opinions on access to medicines. Let’s start.”*

**Theme 1: Availability and Stockouts**

- 1. Can you tell me about the availability of medicines at the health facility closest to you? Are most medicines available? Tell me about which products are often in stock, and which products are not often in stock.
  2. What do you do when products are not in stock?

Probe:

- Do staff tell you where else you can get the medicines? Where do they suggest you should go, and why? What are the advantages and disadvantages to going to this other place?
- Do health providers ever change the prescription or offer a different medicine that is in stock? Can you give me an example of this? Tell me more about what happened. What are the advantages and disadvantages of changing the medicine because the one you needed was not in stock?
- Sometimes health providers will purchase medicines themselves, then sell them to patients if there is a stockout at the facility. Does this sort of thing sometimes happen in the public health facility near you? What is your opinion about this? What do you think are the advantages and disadvantages?
  1. Tell me about how availability of medicines has changed over time.

Probe:

- Were there times when medicines used to be more available than they are now? Can you give me an example? Why do you think it has changed?
- Were there times when medicines used to be less available than they are now? Can you give me an example? Why do you think it has changed?

**Theme 2. Affordability**

- 1. In general, do you feel that medicines are affordable to patients like you? Why or why not?
  2. Do prices for medicines vary in different kinds of facilities? How do they vary? Why do you think they vary?
  3. Can you give an example of when you had to pay, and for what? Where did you get the money to pay? How did you feel about having to pay?
  4. Was there ever a time when you couldn’t afford to pay? What did you do?

Probe:

- Did you have to give up something else you needed in order to pay for medicines? Tell us more about this. What did you give up, and how did you feel about it? What happened next?

- 1. Did you get advice from the provider when you couldn’t afford to pay? What was the advice? Did you follow the advice? What happened next? How did it make you feel?

Probe:

- Told to go to a different facility
- Told to come back a different time
- Told to change treatment or take a different medicine (less expensive)
  1. How does having a chronic illness affect your household expenditures?

Probe:

- Ask about other spending, not just on medicines (travel, companion’s travel, etc.)

2.7 Tell me about how affordability of medicines has changed over time.

Probe:

- Were there times when medicines used to be more affordable than they are now? Can you give me an example? Why do you think it has changed?
- Were there times when medicines used to be less affordable than they are now? Can you give me an example? Why do you think it has changed?

**Theme 3: Quality of medicines**

3.1 In your opinion, how, if at all, does the quality of medicines vary? What are the signs of a good quality medicine?

Probe:

- Describe why you think some medicines have better quality than others.
- Are there differences in quality between locally produced medicines and internationally produced medicines? Or by country
- Are there differences in quality depending on the manufacturer?
- Do you know what a generic medicine is? Is the quality of generic medicines different from brand name medicines?

3.2 Do you think that health providers have perceptions about quality that are similar to yours? Why/why not? If they are different, how?

**Theme 4: Thoughts about other barriers and advice to other patients**

4.1 Are there any other barriers which prevent you from having access to medicines or staying on treatment?

Probe:

- Waiting times
- Adherence problems

4.2 What advice would you give a patient who had just been diagnosed with this chronic illness [the one the patient has]? What would you tell him or her about the best way to get medicines reliably?

Probe:

- Where would you tell them to go?
- What would you tell them in order to not spend too much on medicines?
- What would you tell them to do if they encounter stockouts?
- Any other advice or personal success stories?

**INTERVIEWER: Ask the following question to participants who seem particularly articulate or have very interesting stories about how their lives have been affected by access issues or improved because of better access to medicines.**

4.3 Would you be willing to be contacted again to tell your story?

**INTERVIEWER: “Thank you sincerely for your time. We have completed this interview and are grateful for your help as we collect baseline information in anticipation of this new access to medicines initiative. If in the next couple of days you think of another idea or story to share please beep ” RETURN TO THE COVER PAGE AND NOTE THE TIME THE INTERVIEW WAS COMPLETED.**

**Time Finish** ___________
