# Supplementary material for: Perceptions of Kenyan adults on access to medicines for non-communicable diseases: A qualitative study
Source: PLoS One. 2018 Aug 24;13(8):e0201917. doi: 10.1371/journal.pone.0201917 (PMC6108464; doi:10.1371/journal.pone.0201917)
Supplement: S2 File — (DOCX) [file pone.0201917.s002.docx]

**Instrument ID: H-KI**

Novartis Access Initiative

Household Key Informant Instrument

Hadhira Lengwa:

1. Mtu mzima katika familia aliye na ugonjwa sugu

**Maelezwa kwa mhojaji**

**Hatua ya 1:** **Ridhaa ya Kufahamishwa:** *Omba mshiriki kukupa muda mfupi wa wakati wake. Jitambulishe mwenyewe na utambulishe utafiti. Anza na fomu ya ridhaa ya kufahamishwa kulingana na mafunzo. Ikiwa ridhaa inakubaliwa, mwaachie mshiriki fomu ya ridhaa ya kufahamishwa.*

**Ridhaa ya kufahamishwa ilipokewa kwa mazungumzo ya ana kwa ana?**

**NDIO ________ (endelea na mahojiano)**

**HAPANA**_______ (**SIMAMISHA! Mshukuru mshiriki kwa wakati wake lakini usiendelee na mahojiano)**

***Mhojaji****: Soma maelezo yafuatayo. Tafadhali rudia maelezo haya yakitafsiriwa kwa lugha ya wenyeji kutegemea lugha ya msingi.*

“Asante kwa kukubali kushiriki katika mahojiano haya. Jina langu ni ______. Nitakuwa nikikuuliza maswali. Mwenzangu ______ atakuwa akiandika kumbukumbu.

Tunakusanya deta ya msingi kuhusu upatikanaji wa madawa kwa kutazamia uanzishwaji wa mbinu mpya za kuboresha upatikanaji hivi karibuni. Tunataka kuelewa maoni yako kuhusu upatikanaji wa madawa na nini hutokea wakati madawa hayapatikani. Pia tunependa kukuliza mtazamo wako kuhusu bei na kiwango cha madawa, na jinsi unavyojifunza na kuwafunza wengine kuhusu madawa. Tafadhali uwe huru kutuambia yale unafikiri huna ugumu wa kuyasema. Ukumbuke pia kuwa si lazima useme chochote ambacho unafikiri hufai kukisema. Hakuna majibu yaliyo sahihi na yasiyo sahihi, kwa hivyo tafadhali kuwa mkweli iwezekanavyo ili kutusaidia kuelewa kilicho cha kweli kwako na kwa jamii yako. Uko tayari kuanza?”

**Hatua ya 2***: Tafadhali anzisha mahojiano kwa maswali ya kidemografia.*

**Hatua ya 3:** *Endelea kwa mwongozo wa maswali ya nusu-muundo. Tafadhali peleleza ili kupata kina na undani wa jambo au habari kadiri ya uwezo wako. UKUMBUSHO: Kama kuna ripoti ha hapo kwa hapo kuhusu tukio baya, tafadhali tuma ripoti kwa mtafiti mkuu [Jaza ya habari huhusu njia ya mawasiliano inayofaa kwa mtafiti mkuu au mwakilishi wake hapa]*

**Kitambulisho cha mhojaji** _________ **Kitambulisho cha mwandishi wa kumbukumbu ________**

1. **Tarehe ya mahojiano (SS/MM/MMMM)** __________________
2. **Muda wa kuanza _**______ **Muda wa kumaliza _**_______________
3. **Herufi za jina la Msimamizi __________**

**Table 1: Demografia ya mhojiwa**

***Mhojaji:*** *“Nitaanza kwa kukuuliza maswali kukuhusu wewe mwenyewe.”*

| Q# | SWALI | KODI | | Jibu | RUKA |
| --- | --- | --- | --- | --- | --- |
| 000. | Mhojiwa/Aina ya ala | F-KI: **(1)** | | **1**  _____________ |  |
|  | Jinsia ya Mhojiwa | Mwanaume  **(1)**  Mwanamke (**2)** | | _____________ |  |
|  | Umri |  | | _____________ |  |
| 102. | Nambari ya simu |  | | ____________ | |
| 103. | Jina la kituo cha afya cha karibu |  | | ____________ | |
|  | Programu ya *Novartis Access* imesanifiwa ili kuongeza upatikanaji na uwezo wa ununuzi wa madawa ya kiwango cha juu ya kutibu magonjwa mbalimbali sugu.  Umewahi kusikia kuhusu *Novartis Access* kabla ya sasa? | | Hapana (**0)**  Ndio (**1)** | __________ |  |

**Maelezo kuhusu mahojiano ya nusu-muundo: *Mhojaji:*** *“Sasa nitakuuliza maswali kuhusu maoni na mawazo yako kuhusu upatikanaji wa madawa. Tuanze.”*

**Mada ya 1: Kupatikana na Kukosekana**

- 1. Unaweza kuniambia kuhusu upatikanaji wa madawa katika kituo cha afya cha umma kilicho karibu na wewe? Madawa mengi yanapatikana? Niambie mara nyingi ni madawa gani yanapatikana, na mara nyingi ni madawa gani hayapatikani.
  2. Huwa unafanya nini kama madawa na bidhaa hazipatikani?

Chunguza:

- Huwa wafanyakazi wanakwambia mahali pengine unaweza kupata madawa? Huwa wanakupendekezea uende wapi, na kwa nini? Kuna manufaa na hasara gani kutokana na kwenda kununua madawa kutoka mahali huku kwingine?
- Wahudumu wa afya wanaweza kukuandikia madawa tofauti au kukupa dawa tofauti ambayo bado inapatikana kwenye bohari? Unaweza kunieleza kwa kutoa mfano? Niambie zaidi kuhusu kilichotendeka. Kuna manufaa gani na hasara gani kutokana na kubadilishiwa dawa kwa sababu dawa uliyohitaji imekwisha?
- Wakati mwingine wanaotoa huduma za afya hununua madawa wao wenyewe, na kuyauza kwa wagonjwa kama madawa yamekwisha katika kituo cha afya. Kuna wakati mwingine jambo hili hufanyika katika kituo cha afya kilicho karibu nawe? Nini maoni yako kuhusu jambo hili? Unafikiri kuna faida gani na hasara gani?
  1. Niambie jinsi upatikanaji wa madawa umebadilika kulingana na mpito wa wakati.

Chunguza:

- Kulikuwa na wakati ambapo madawa yalikuwa yanapatikana kuliko yanavyopatikana sasa? Unaweza kunipa mfano? Kwa nini unafikiri kumetokea mabadiliko ya aina hii?
- Kulikuwa na wakati ambapo madawa yalikuwa hayapatikana kama yanavyopatikana sasa? Unaweza kunipa mfano? Kwa nini unafikiri hali imebadilika?

**Mada ya 2. Unafuu**

- 1. Kwa jumla, unafikiri bei ya madawa ni nafuu kwa wagonjwa kama wewe? Kwa nini ndivyo au kwa nini sivyo?
  2. Bei za madawa huwa tofauti kutoka kituo kimoja cha afya hadi kingine? Huwa tofauti kivipi? Kwa nini unafikiri bei huwa tofauti?
  3. Unaweza kunipa mfano wa wakati ulilazimika kulipa na ulilipia nini? Ulitoa wapi pesa za kulipa? Ulijisikiaje kuhusu kulazimika kulipa?
  4. Kumekuwa na wakati ambapo ulishindwa kulipia dawa? Ulifanya nini??

Chunguza:

- Uliwahi kukosa kununua kitu kingine ulichohitaji ili kulipia madawa? Niambie zaidi kuhusu tukio hili. Ulikosa kununua au kufanya nini, na ulifikiri nini kuhusu jambo hilo? Nini kilifuata?

- 1. Ulipata ushauri kutoka kwa watoa huduma wakati uliposhindwa kulipia dawa? Ulishauriwa nini? Ulifuata ushauri huo? Ulifanya nini baada ya hapo? Jambo hili lilikufaya ujisikieje?

Chunguza:

- Kuambiwa uende katika kituo kingine
- Kuambiwa urudi tena wakati mwingine tofauti
- Kuambiwa ubadilishe matibabu au kutumia madawa tofauti (yasiyo ghali)
  1. Kwa njia gani kuwa na ugonjwa sugu kumeathiri matumizi ya pesa nyumbani mwako?

Chunguza:

- Uliza kuhusu gharama nyingine, sio tu kwa madawa (usafiri, usafiri wa mwenzi, n.k.)

2.7 Niambie jinsi unafuu wa madawa umebadilika kulingana na mabadiliko ya wakati.

Chunguza:

- Kulikuwa na wakati bei ya madawa ilikuwa nafuu kuliko ilivyo sasa? Unaweza kunipa mfano? Kwa nini unafikiri kuna mabadiliko?
- Kulikuwa na wakati bei ya madawa ilikuwa ghali kuliko ilivyo sasa? Unaweza kunipa mfano? Kwa nini unafikiri kuna mabadiliko?

**Mada ya 3: Ubora wa madawa**

3.1 Kwa maoni yako, kwa vipi, kama kuna uwezekano, ubora wa madawa hutofautiana? Ni alama gani huonyesha kuwa dawa Fulani ni bora?

Chunguza:

- Eleza kwa nini unafikiri madawa fulani ni bora kuliko mengine.
- Kuna tofauti katika ubora kati ya madawa yanayotengenezwa nchini na yale yanaagizwa kutoka nchi za kigeni? Au kutegemea nchi?
- Kuna tofauti ya ubora kutegemea shirika la ketengeneza madawa?
- Unajua ni madawa gani yasiyo na majina ya chapa? Je, unafikiri ubora wa madawa yasiyo na majina ya chapa ni tofauti na madawa yaliyo na majina ya chapa?

3.2 Je, unafikiri kuwa wanaotoa huduma za afya wana mtazamo kuhusu ubora ulio sawa na mtazamo wako? Kwa nini/kwa nini sivyo? Kama ni tofauti, kwa njia gani?

**Mada ya 4: Fikira na mawazo kuhusu vizuizi vingine na ushauri kwa wagonjwa wengine**

4.1 Kuna vizuizi vingine vinavyokukosesha kuwa na uwezekano wa kupata madawa au kuendelea na matibabu?

Chunguza:

- Kipindi cha kusubiri au kungojea
- Matatizo ya uzingatiaji wa maagizo

4.2 Ni ushauri gani ungempa mgonjwa ambaye ametambuliwa kuwa na ugonjwa sugu kwa mara ya kwanza [ugonjwa mgonjwa alio nao]? Ungemwambia nini kuhusu njia gani nzuri ya kupata madawa bila kukosa?

Chunguza:

- Ungemshauri aende wapi?
- Ungemshauri nini ili asitumie kiasi kikubwa cha pesa katika kununua madawa?
- Ungemshauri aende wapi ikiwa madawa yamekwisha?
- Ushauri mwingine wowote au habari yoyote kuhusu kufanikiwa kwako?

**MHOJAJI: Uliza swali lifuatalo kwa washiriki wanaoonekana kuwa wenye ufasaha au wenye habari za kusisimua kuhusu jinsi maisha yao yameathiriwa kwa ajili ya upatikanaji wa madawa au yameimarika kutokana na ubora wa upatikanaji wa madawa.**

4.3 Je, ungependa kupokea mawasiliano tena kutoka kwetu ili uweze kutupa maelezo zaidi kuhusu maisha yako?

**MHOJAJI: “Asante sana kwa muda wako. Tumekamilisha mahojiano haya na tunakushukuru kwa msaada wako tunapoendelea kukusanya deta ya awali tukitarajia mbinu mpya ya kuwezesha upatikanaji wa madawa kwa urahisi. Ikiwa kwa siku mbili zijazo unafikiri kuhusu mawazo mapya au habari ya kueleza tafadhali wasiliana nasi” RUDI KWENYE UKURASA WA MBELE NA KUANDIKA MUDA MAHOJIANO HAYA YAMEKAMILIKA.**
